# Myeloproliferative Syndrome With Eosinophilia Associated With Translocation t(8; 13) and T-cell Lymphoblastic Lymphoma: A Case Report and Review of the Literature

**DOI:** 10.7759/cureus.22734

**Published:** 2022-03-01

**Authors:** Lizeth Yamile Urrea Pineda, Oliver Perilla, Vanessa Santiago-Pacheco, Simon Trujillo Montoya

**Affiliations:** 1 Internal Medicine, Hospital San Vicente Fundacion, Rionegro, COL; 2 Hematology, Hospital San Vicente Fundacion, Rionegro, COL; 3 Pathology, Universidad de Antioquia, Medellín, COL; 4 Internal Medicine, Hospital Sanvicente Fundacion, Rionegro, COL

**Keywords:** fibroblastic growth factor receptor-1 gene (fgfr1), eosinophilia, lymphadenopathy, myeloid leukemia, precursor t-cell lymphoblastic leukemia-lymphoma

## Abstract

The 8p11 myeloproliferative syndrome (EMS) is an aggressive neoplasm associated with chromosomal translocations involving the fibroblast growth factor receptor 1 tyrosine kinase gene on chromosome 8p11. We report the case of a 31-year-old man with no prior medical history who presents with two weeks of sore throat and cervical lymphadenopathy up to 5 cm. Initial peripheral blood examination showed leukocytosis with predominantly neutrophils and eosinophilia. A CT scan demonstrated mediastinal lymphadenopathies, liver enlargement and splenomegaly. An excisional biopsy of a cervical lymph node demonstrated findings consistent with a diagnosis of T-cell lymphoblastic lymphoma. Bone marrow aspirate and biopsy revealed hypercellular marrow with granulocytic predominance, left-shifted granulopoiesis, eosinophilia and the cytogenetic analysis showed the following karyotype: 46, XY, t(8;13). The final diagnosis was a myeloproliferative syndrome with eosinophilia related to t(8;13) and T-cell acute lymphoblastic lymphoma (8p11 myeloproliferative syndrome). We review the relevant literature about this unusual entity.

## Introduction

8p11 myeloproliferative syndrome is a rare hematologic malignancy characterized by the presence of myeloid proliferation, eosinophilia and lymphoblastic lymphoma associated with balanced translocations involving chromosome 8p11, mainly t(8;13) (p11;q12). In 2016 the World Health Organization (WHO) classified this disease in the category of myeloid/lymphoid neoplasms associated with eosinophilia with Eosinophilia-myalgia syndrome (FGFR1) rearrangement [1].

The translocation results in the creation of a novel ZNF198-FGFR1 fusion protein and constitutive activation of the FGFR1 tyrosine kinase, activating multiple signaling pathways that result in cell transformation. This is a rare disease with very few patients reported around the world and it can be found at any age with a slightly male-to-female predominance [2,3]. Patients usually present with lymphadenopathy, leukocytosis with left shift with predominant eosinophilia in peripheral blood and/or bone marrow. 

Most of the patients show a hypercellular bone marrow that leads to a diagnosis of myeloid hyperplasia or a myeloproliferative neoplasm. In lymph node biopsies the most reported finding is a T-cell lymphoblastic lymphoma [2]. Numerous and varied therapeutic regimens have been used for patients with EMS but overall these therapies have shown unsatisfactory results. This aggressive disease has a high rate of progression to acute myeloid leukemia resistant to conventional chemotherapy. At this moment, stem cell transplant remains the only possibility for long-term survival [2,4].

## Case presentation

A 31-year-old man presented to a local hospital with two weeks of odynophagia and painful bilateral cervical and submandibular lymphadenopathy. He was diagnosed with acute pharyngotonsillitis and received treatment with a short course of oral antibiotics and NSAIDs (non-steroidal anti-inflammatory drugs) without improvement so he was admitted to Hospital San Vicente Fundación Rionegro. He had no fever or other relevant symptoms and no pathological, toxic, expositional or epidemiological past medical history. Physical examination revealed enlarged tonsils with white exudate. Additionally, he had mobile and painless cervical and submandibular lymphadenopathy up to 5 cm, without any palpable lymphadenopathies in any other location. Infectious mononucleosis was considered but serological tests for Epstein-Barr virus, toxoplasma, cytomegalovirus and human immunodeficiency virus were negative. A CT scan demonstrated mediastinal lymphadenopathies, liver enlargement and splenomegaly. An excisional biopsy of a cervical lymph node was performed for histopathological and microbiological studies.

The initial complete blood analysis revealed leukocytosis with left shift (total leukocytes: 43,800 x mm^3^, neutrophils: 28,500 x mm^3^, lymphocytes: 3,500 x mm^3^, myelocytes: 5,000 x mm^3^, metamyelocytes: 3,000 x mm^3^, eosinophils: 4,400 x mm^3^) and polycythemia (hemoglobin concentration 20 g/dL). Bone marrow cytology smears and biopsy was performed.

**Figure 1:**
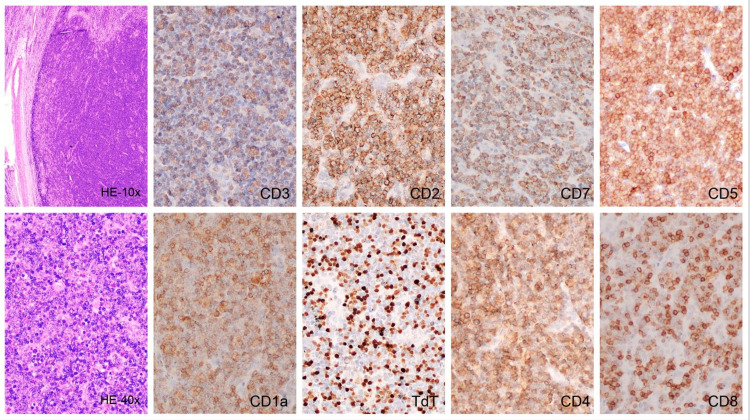
Cervical lymph node, infiltrated by small to medium atypical lymphoid cells with scattered eosinophils (H&E x100, H&E x400). Immunohistochemistry shows partial loss of CD3 expression (x400), and positive staining for CD2 (x400), CD5 (x400), CD7 (x400), CD1a (x400), TdT (x400), CD4 (x400), CD8 (x400).

The cervical lymphadenopathy biopsy demonstrated loss of nodal architecture associated with a diffuse and vaguely nodular proliferation of medium to small-sized cells, accompanied by scattered eosinophils, with the following immunophenotype: dim CD3, CD7 (+), CD5(+), CD4 (+), CD8 (+), CD2 (+), TdT (+), CD34 (-), CD1a (+), Perforin (-), CD30 (-), Ki67 of 70%-80%, consistent with a diagnosis of T-cell lymphoblastic lymphoma (T-LBL) (Figure 1).

**Figure 2:**
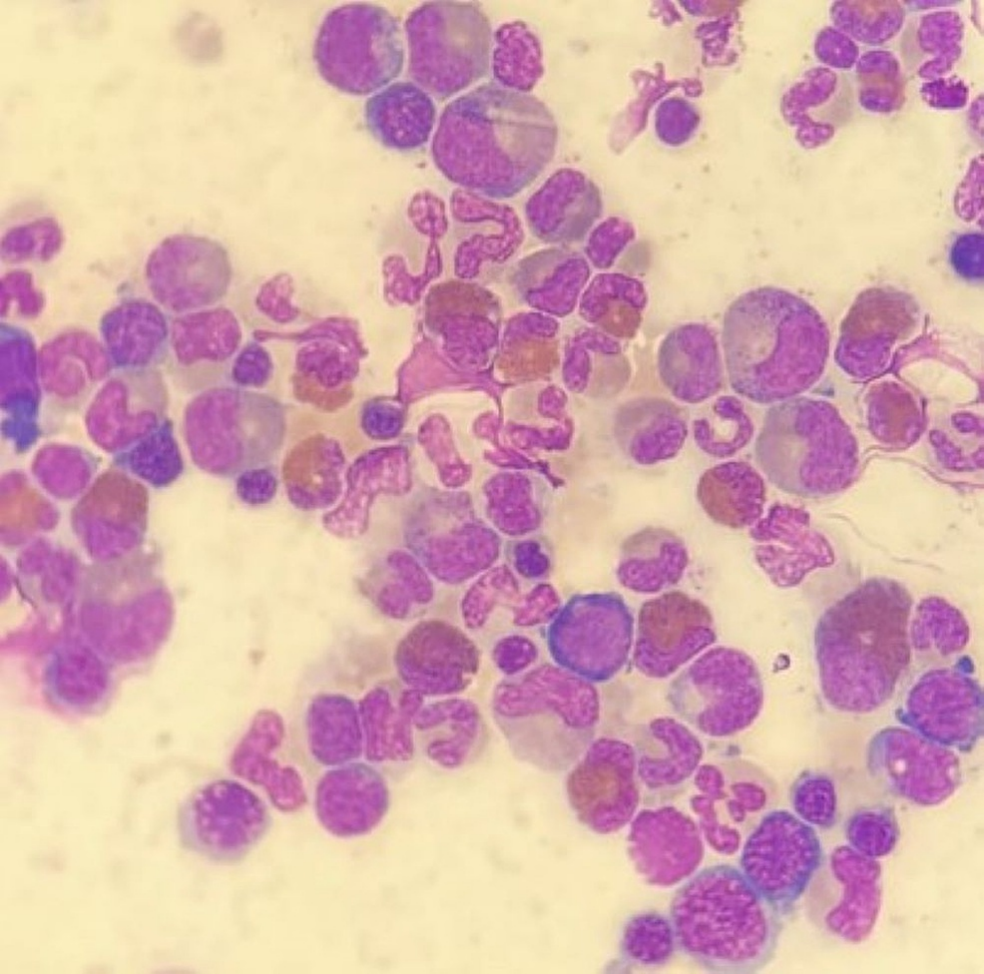
Bone marrow aspirate revealed a hypercellular marrow with markedly increased eosinophilic precursors, predominantly mature forms; mildly increased granulocytes without morphologic dysplasia; no overt increase in blasts was found.

**Figure 3:**
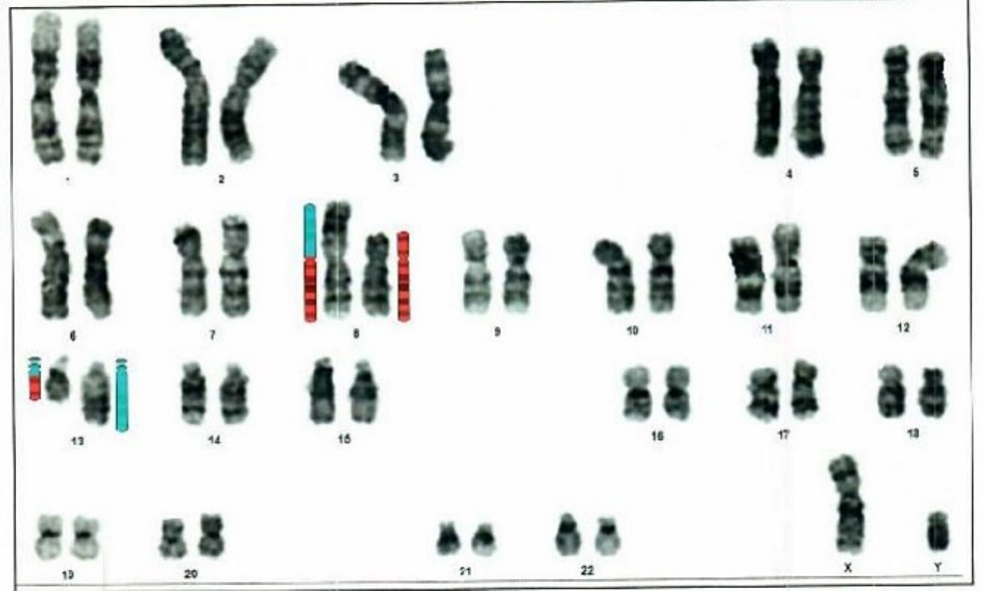
Karyotype: 46,XY,t(8;13)(p11.2:q13)(34). Cytogenetic analysis of tissue, with the chromosome banding technique used, revealed the exchange of genetic material between the short arm of chromosome 8 and the long arm of chromosome 13 in all metaphases.

Bone marrow aspirate and biopsy showed hypercellular marrow with granulocytic predominance, left-shifted granulopoiesis, eosinophilia (20%) and the blast count were not increased (Figure 2). Cytogenetic analysis showed the following karyotype: 46, XY, t(8;13) (Figure 3). FISH for C-MYC (8q;14q) as well as PCR for BCR/ABL p210 in peripheral blood were negative. A diagnosis of chronic myeloproliferative syndrome with eosinophilia associated with FGFR1 gene translocation was made. GRAALL-2003 chemotherapy was initiated (high doses of prednisone, vincristine, and asparaginase in combination with daunorubicin, cyclophosphamide, and intrathecal methotrexate) and completed all consolidation cycles including holocephalic radiotherapy. He relapsed at the beginning of maintenance therapy and was switched to HyperCVAD with an increased doses regimen (hyperfractionated cyclophosphamide, vincristine, doxorubicin and dexamethasone) obtaining a second complete hematological response. Afterward, the patient underwent allogeneic transplantation of hematopoietic precursors and achieved complete remission.

 

## Discussion

The term 8p11 myeloproliferative syndrome was initially suggested by Macdonald et al. in 1995, who described a syndrome with common clinical features that met the following criteria [4,5]: (1) Myeloproliferative neoplasm associated with peripheral eosinophilia, (2) lymphadenopathy usually involved by non-Hodgkin's lymphoma/leukemia of T lineage, (3) frequent progression to acute myeloid leukemia, and (4) reciprocal translocations involving chromosome 8p11 [2]. Case reports with the described characteristics have been previously published [6-8]. The 8p11 myeloproliferative syndrome was included in the latest WHO 2016 review of myeloid neoplasms and acute leukemia within the category of myeloid/lymphoid neoplasms associated with eosinophilia with FGFR1 rearrangement [1,9]. This syndrome affects patients in a wide age range (3-84 years) [2,10]. and usually present with lymphadenopathy (75%), leukocytosis with left shift (92%) with predominant eosinophilia in peripheral blood and/or bone marrow (79% and 71%, respectively). They may have splenomegaly (58%) and hepatomegaly (42%). Most of them present with constitutional symptoms at the time of diagnosis but approximately 20% are asymptomatic [2]. The average amount of time patients experience symptoms prior to diagnosis is two years.

Most case reports describe bone marrow involvement due to myeloid hyperplasia with or without eosinophilia and up to 55% may have blasts in variable percentages (approximately 16% meet morphologic criteria for acute myeloid leukemia). Most lymph node biopsies demonstrate T-cell lymphoblastic lymphoma involvement (79%), but cases of bilineal lymphomas (mixed myeloid/lymphoid component) and less frequently myeloid sarcoma (21%) or B-cell lymphoblastic lymphoma have also been reported [2,11].

In 2008, Vega et al. reported the histological and immunophenotypic characteristics of lymph node biopsies from six cases of EMS associated with t(8,13). They describe the coexistence of two cellular components in the same site: myeloblasts and lymphoblasts. Myeloblasts tend to surround lymphoid follicles and/or blood vessels, accompanied by numerous scattered eosinophils.

Lymphoblasts are of usual immunophenotype, mostly immature T type (common expression of TdT, CD1a and pan-phenotypic T-cell markers); in contrast, the myeloblastic component show expression of at least some immunophenotypic marker of myeloid lineage (CD15, CD68, CD117, lysozyme or other myeloid type antigens). Interestingly, in this report, both cell types had a CD3 expression, suggesting that these lymphomas are bilineal [10]. In the case of our patient, the lymph node findings were compatible with T-cell lymphoblastic lymphoma with numerous eosinophils, but unfortunately, we had no report of other markers for myeloid lineage in the lymph node histology. 

Karyotype analysis has been the most commonly used diagnostic method to identify the 8p11 translocation. All translocations and insertions involving FGFR1 are visible by this method. FISH tests have shown that t(8,13) is not only reciprocal but can also involve inversion of 13q11-12 and have also demonstrated that this translocation is present in both immature myeloid cells and lymphoblasts. The variant translocations or insertions are shown in Table 1 [2].

**Table 1:** Clinicopathologic correlation with variants of 8p11 myeloproliferative syndrome Adapted from Reference [4].

Karyotype	Clinicopathologic association
t(8;13)(p11;q12)	Lymphadenopathy
t(8;9)(p11;q33)	Peripheral blood monocytosis Tonsillar enlargement
t(6;8)(q27;p11-12)	Older age, median 61 y Polycythemia, peripheral blood eosinophilia
t(8;22)(p11;q11)	Basophilia

Molecular tests demonstrate that all cases have a chromosomal abnormality involving translocation or insertion of the FGFR1 gene located on chromosome 8p11 with other genes. The most frequent rearrangement is with the zinc finger gene ZNF198 in chromosome 13q12 t(8,13) (ZNF198- FGFR1) which in turn generates a constitutively activated fusion protein tyrosine kinase type FGFR1 with the ability to transform murine hematopoietic cell lines through activation of effector pathways such as STAT1, STAT5, PI3K, PLC and MAP kinase [12,13]. To date, there have been 13 translocations and one insertion involving chromosome 8p11 identified. Patients with other translocations involving FGRF1 may have different clinicopathologic features; for example, those with t(8,22) have a leukocytosis with basophilia rather than eosinophilia (a pattern similar to chronic myeloid leukemia) and generally do not present with lymphadenopathy [2].

The clinical manifestations of EMS are variable and depend in part on whether or not the neoplasm is in the chronic or acute phase. The differential diagnosis is wide and includes myeloproliferative neoplasms mainly in the chronic phase, acute myeloid leukemia, acute lymphoblastic leukemia (acute phase) [2].

Findings associated with hypereosinophilias such as lymphadenopathy, leukocytosis with a left deviation and the absence of reactive causes increase the clinical suspicion of clonal eosinophilia associated with a hematopoietic neoplasm [12] Peripheral blood smears should be screened for blasts, monocytosis, basophilia, leukocytosis with a left deviation, dysplasia or leukoerythroblastosis, which have been associated with the MPN or MDS/MPN phenotypes of rearranged PDGFRA/B, FGFR1 and JAK [13].

The clinical course of this disease is aggressive, with a high risk of progression to acute myeloid leukemia at two-year follow-up and less frequently, to acute lymphoblastic leukemia. Numerous and varied therapeutic regimens have been used for patients with EMS including protocols for acute lymphoblastic leukemia, AML and myeloproliferative neoplasms. Overall, these therapies have shown unsatisfactory results. To date, the few long-term survivors of EMS are those who have undergone stem cell transplants. Follow-up treatment data for patients with myeloproliferative syndrome 8p11 show that only 27% achieved complete clinical response, 45% partial response and 28% had no response [2]. Overall survival is 15 months and patients usually progress to chemotherapy-resistant acute myeloid leukemia. Allogeneic hematopoietic stem cell transplantation (HSCT) is the only curative option. Tyrosine kinase inhibitors such as Ponatinib can reduce the number of FGFR1-positive colonies, but there are no clinical studies supporting their use [14,15].

## Conclusions

The 8p11 myeloproliferative syndrome is an infrequent and aggressive neoplasm associated with chromosomal translocations involving the fibroblast growth factor receptor 1 tyrosine kinase gene. Patients often present with peripheral blood eosinophilia and lymphadenopathy. The natural history of this neoplasm is to evolve into acute leukemia, usually of myeloid or mixed lineage. The prognosis is poor despite aggressive chemotherapy, with a few patients achieving long clinical remission after stem cell transplantation.
